# Tract‐specific analysis and neurocognitive functioning in sickle cell patients without history of overt stroke

**DOI:** 10.1002/brb3.1978

**Published:** 2021-01-12

**Authors:** Yaqiong Chai, Chaoran Ji, Julie Coloigner, Soyoung Choi, Melissa Balderrama, Chau Vu, Benita Tamrazi, Thomas Coates, John C. Wood, Sharon H. O'Neil, Natasha Lepore

**Affiliations:** ^1^ CIBORG Laboratory Department of Radiology Children's Hospital Los Angeles Los Angeles CA USA; ^2^ Department of Radiology Children's Hospital Los Angeles Los Angeles CA USA; ^3^ Department of Biomedical Engineering University of Southern California Los Angeles CA USA; ^4^ Department of Electrical Engineering University of Southern California Los Angeles CA USA; ^5^ Division of Cardiology Children's Hospital Los Angeles Los Angeles CA USA; ^6^ Neuroscience Graduate Program University of Southern California Los Angeles CA USA; ^7^ Department of Pediatrics Keck School of Medicine University of Southern California Los Angeles CA USA; ^8^ Division of Hematology, Oncology, and Blood and Marrow Transplantation Children's Hospital Los Angeles Los Angeles CA USA; ^9^ Division of Neurology Children's Hospital Los Angeles Los Angeles CA USA; ^10^ The Saban Research Institute Children's Hospital Los Angeles Los Angeles CA USA

**Keywords:** microstructural damage, neurocognitive, sickle cell disease, tract‐specific analysis, white matter

## Abstract

**Introduction:**

Sickle cell disease (SCD) is a hereditary blood disorder in which the oxygen‐carrying hemoglobin molecule in red blood cells is abnormal. SCD patients are at increased risks for strokes and neurocognitive deficit, even though neurovascular screening and treatments have lowered the rate of overt strokes. Tract‐specific analysis (TSA) is a statistical method to evaluate microstructural WM damage in neurodegenerative disorders, using diffusion tensor imaging (DTI).

**Methods:**

We utilized TSA and compared 11 major brain WM tracts between SCD patients with no history of overt stroke, anemic controls, and healthy controls. We additionally examined the relationship between the most commonly used DTI metric of WM tracts and neurocognitive performance in the SCD patients and healthy controls.

**Results:**

Disruption of WM microstructure orientation‐dependent metrics for the SCD patients was found in the genu of the corpus callosum (CC), cortico‐spinal tract, inferior fronto‐occipital fasciculus, right inferior longitudinal fasciculus, superior longitudinal fasciculus, and left uncinate fasciculus. Neurocognitive performance indicated slower processing speed and lower response inhibition skills in SCD patients compared to controls. TSA abnormalities in the CC were significantly associated with measures of processing speed, working memory, and executive functions.

**Conclusion:**

Decreased DTI‐derived metrics were observed on six tracts in chronically anemic patients, regardless of anemia subtype, while two tracks with decreased measures were unique to SCD patients. Patients with WMHs had more significant FA abnormalities. Decreased FA values in the CC significantly correlated with all nine neurocognitive tests, suggesting a critical importance for CC in core neurocognitive processes.

## Graphical abstract

Sickle cell disease (SCD) is a genetic blood disorder with high prevalence of silent stroke and white matter damage. We utilized tract‐specific analysis and compared 11 major brain WM tracts between SCD patients and healthy controls, in correlation processing speed and response inhibition. Our results implicate a relationship between microscopic damage to WM structural damage and neurocognitive function in SCD patients.

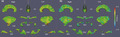

## 1 INTRODUCTION

Sickle cell disease (SCD) is an inheritable genetic disorder of red blood cells, in which a single base pair DNA mutation causes hemoglobin to polymerize upon deoxygenation, producing sickle‐shaped red blood cells. It is a major public health concern, with over 300,000 children born with SCD each year worldwide, with incidence rates projected to increase to 400,000 by 2050 (Piel et al., 2013). Individuals living with the disease experience lifelong complications, including anemia, infections, stroke, tissue damage, organ failure, pain crises, and premature death (King et al., 2014; Rees et al., 2010).

While SCD affects many vital organs, damage to brain tissue is among the most concerning due to the profound personal, professional, and social cost to patients (Adams et al., 2002; Debaun & Kirkham, 2016). Until recently, ten percent of children with SCD had a symptomatic, overt stroke, with incidence rates rising to 24% by 45 years of age (Ohene‐Frempong et al., 1998), though in the last 16 years, routine transcranial Doppler (TCD) screening of the Circle of Willis and chronic transfusion therapy when indicated have lowered the risk tenfold (Adams et al., 1998; Bernaudin, Verlhac, Arnaud, Kamdem, Chevret, et al., 2014). However, while the risk for silent cerebral infarcts (SCI) is also reduced by transfusion, SCI remain a serious concern due to associated neurocognitive deficits in comparison to lesion‐free patients (Debaun et al., 2012, 2014). Approximately 27% of children experience SCI by 6 years of age and 37% by 14 years of age, with ongoing risk of progressive injury, with the size and number of lesions increasing with age(Bernaudin, Verlhac, Arnaud, Kamdem, Vasile, et al., 2014), reaching up to 53% by 30 years of age (Kassim et al., 2016).

In addition to SCIs, which primarily damage the white matter (WM) in the brain, SCD patients also show reduced WM volume relative to controls (Choi et al., 2017, 2019; Steen et al., 2005). These abnormalities may lead to affective and cognitive consequences over time, including depression and isolation due to physical and mental limitations, as well as poor neurocognitive outcomes (Daniel Armstrong et al., 1996; Prussien et al., 2018; Schatz et al., 2001). In adolescent SCD patients, a range of academic and neurocognitive difficulties compared to healthy peers has been reported, including lower verbal IQ scores, poorer math performance, and impairments in visual‐motor functions (Prussien et al., 2019). Additionally, there is a preponderance of evidence that individuals with SCD experience difficulties with working memory, executive functions and processing speed (Stotesbury, 2018; Kral et al., 2006; van der Land et al., 2015), neurocognitive domains sensitive to WM compromise (Jacobs et al., 2013; Turken et al., 2008).

Brain magnetic resonance imaging (MRI) is commonly used for the detection of cerebrovascular damage. While clinical imaging protocols of MRI typically rely on qualitative assessments of T1 and T2‐weighted images, diffusion tensor imaging (DTI) has proven to be a more sensitive technique to probe WM microstructure, characterize abnormalities of WM pathways in various neurological disorders (Sundgren et al., 2004), and to detect preclinical neurologic ischemia (Basser et al., 1994). Previous studies have used methods such as region of interest (ROI) analysis (Balci et al., 2012), voxel‐based analyses as in voxel‐based morphometry (VBM) (Baldeweg et al., 2006), and WM tract‐based methods such as Tract‐Based Spatial Statistics (TBSS) (Kawadler et al., 2015; Smith et al., 2006) and Tract‐Specific Analysis (Chai et al., 2015; Zhang et al., 2010), of which the latter two are WM tract‐based methods. Balci et al. (2012), the first to use DTI in SCD cohorts, reported significantly reduced fractional anisotropy (FA) values and increased apparent diffusion coefficient values in the corpus callosum (CC) and cortico‐spinal tracts (CST) using an ROI‐based analytic approach. Likewise, Sun et al. (2012) used TBSS and found similar outcomes in both the CC and CST for asymptomatic SCD patients. Nevertheless, few studies have systematically explored microscopic regional WM changes in relation to neurocognitive performance in patients with SCD (Daniel & Pegelow, 2016), but exploring this relationship may inform SCI prevention and early intervention efforts.

Although TBSS is a widely used tool, TSA has several advantages for examining WM differences in specific tracts across populations. TBSS lacks anatomical specificity because it constructs the skeleton for the entire WM, instead of separately for each individual WM tract, like TSA. TSA also makes it possible to distinguish between adjacent WM tracts, such as the CC and CST, two of the most prominent WM tracts. In TSA, WM fibers are segmented on a population‐specific template, and statistical comparisons are performed on DTI‐derived measurements projected on a medial sheet generated from these tracts. By focusing on specific tracts, it provides better localization of areas affected by neurological disorders. Additionally, recent studies comparing congenitally blind versus sighted control groups (Lao et al., 2015), investigating 22q11.2 deletion syndrome (Yushkevich et al., 2008) and preterm infants (Pecheva et al., 2017), suggest that TSA can outperform TBSS in terms of statistical detection power.

In this study, we utilize TSA to explore differences in major brain WM tracts: corpus callosum, right and left cortico‐spinal tracts, inferior fronto‐occipital fasciculus (IFO), inferior longitudinal fasciculus (ILF), superior longitudinal fasciculus (SLF), and uncinate fasciculus (UNC). The studied groups are as follows: patients with SCD, anemic controls with normal hemoglobin, and healthy controls. We additionally examine performance on measures of processing speed, working memory and executive functions, as well the relationship between the most commonly used DTI metric of WM tracts and neurocognitive performance in the SCD patients and healthy controls.

## 2 METHODOLOGY

### 2.1 Participants

Following Institutional Review Board at Children's Hosptial Los Angeles (CHLA), all participants provided consent or assent. Patients and controls were recruited from the CHLA patient population, their families and the community. Study participants completed a 75‐min MRI examination without sedating medications, limiting our study to subjects older than 10 years of age. Exclusion criteria for SCD patients included previous overt stroke, significant cerebral vasculopathy on previous imaging studies, acute chest syndrome, pregnancy, and pain crisis hospitalization within 1 month of the study. SCD patients at CHLA are regularly screened with TCD ultrasonography to identify patients at higher risk of stroke. Patients with a blood flow velocity faster than 200 cm/s read from TCD are placed on monthly blood transfusions to suppress the percentage of hemoglobin S to less than 30%, in accordance with standard clinical practice.

The healthy control group (CTL) consisted of subjects with no known chronic medical conditions, prior history of neurologic insults, or developmental delay. CTL subjects were recruited from friends and family of the SCD patients to better match for ethnicity, socioeconomic status, and other environmental factors. To control for possible changes related to anemia, independently of sickle hemoglobin, we also recruited patients with non‐sickle chronic anemia syndromes (ACTL), including beta‐thalassemia major, beta‐thalassemia intermedia, and congenital dyserythropoietic anemia. Some of the patients in the SCD and ACTL cohort were on chronic transfusion. For these patients, MRI examination was performed prior to a regularly scheduled transfusion to better match the hematocrit to the non‐transfused anemic patients.

### 2.2 Neuroimaging

Each subject underwent a MRI study using a 3T Philips Achieva with an 8‐element array coil. Whole brain T2‐FLAIR images were acquired to screen for WM hyperintensities. Acquisition parameters were as follows: TR = 4,800 ms, TE = 363 ms, FOV = 256 × 256 mm, and voxel size of 1.3 × 1 × 1 mm. T2‐FLAIR images were read for WM abnormalities by a board certified neuroradiologist, who was blinded to disease status. White matter hyperintensities (WMHs), indicating SCIs, were classified as 3–5 mm lesions on T2‐FLAIR, observed in two orthogonal planes. These lesions had no known neurological sequelae. Diffusion‐weighted images (DWI) were obtained in 30 encoding directions at a *b*‐value of 1,000 m/s^2^, and a reverse gradient *b* = 0 using a single‐shot echo‐planar imaging sequence (TR = 6,700 ms, TE = 86 ms, FOV = 240 × 240 mm, voxel size of 2.5 × 2.5 × 2.5 mm).

### 2.3 Registrations and 3D representations

DWI data were visually inspected for major artifacts and signal drop off. The 4‐D images were corrected for eddy‐current introduced geometric distortions using the Tensor Toolkit (TTK), as part of the ITK software package (www.itk.org). In addition, we applied susceptibility‐induced off‐resonance field corrections by calculating field maps from reverse gradient *b* = 0 images using TOPUP (Andersson et al., 2003). DWI images were skull‐stripped using DSI studio (http://dsi‐studio.labsolver.org ), followed by tensor estimation using TTK.

WM microstructural differences were compared in atlas space. The atlas in the current context is a study‐specific DTI template constructed from healthy adults, that is, a volume that captures the average shape and diffusion‐based features of the entire cohort (Zhang et al., 2017). We conducted rigid, affine, and non‐linear registration using Diffusion Tensor Imaging Toolkit (DTI‐TK) (Zhang et al., 2006) to align tensor images to the atlas space. We did not use scalar‐based images such as FA because they discard orientation information, making it difficult to distinguish neighboring tracts with similar FA values but different orientations. Additionally, registering tensor images onto a template yields better alignment of the dominant diffusion orientation (Yushkevich et al., 2008). Rigid alignment was employed first to find an initial linear estimation of the original image in the template space. Affine alignment and a non‐linear registration were performed using a deformation field map to improve alignment quality (Zhang et al., 2007).

Tracts of interest were generated as binary segmentation using deterministic tractography (Lawes et al., 2008). Then a continuous medial representation (cm‐rep) model (Yushkevich et al., 2006), a deformable modeling and shape analysis technique, was formulated and a medial surface was approximated for each vertex, so that the skeleton and boundary were defined simultaneously for each tract (Yushkevich, 2009). Diffusion data from every subject were then projected onto the skeleton, by searching along the unit normal from the vertex to the tract boundary, which defines the stopping criteria (Yushkevich et al., 2008). This sampling strategy limits potential voxel mis‐assignments from neighboring tracts. Maximum or mean value of the DTI metrics can be projected and sampled (Zhang et al., 2009). Statistical analysis of the projected diffusion metrics at each vertex on the skeleton is given in the following sections.

**FIGURE 1:**
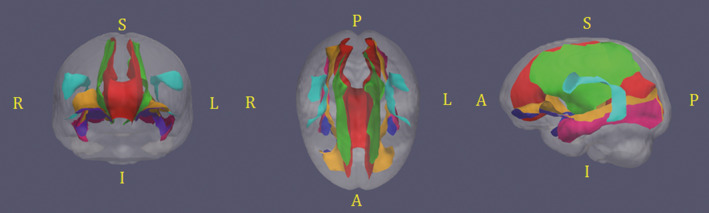
Medial representation of the 11 white matter tracts in coronal, axial, and sagittal view. Red: corpus callosum (CC), green: cortico‐spinal tract (CST), orange: inferior fronto‐occipital tracts (IFO), magenta: inferior longitudinal tracts (ILF), cyan: superior longitudinal tracts (SLF) and purple uncinates (UNC)

In this work, maximum FA values were computed locally and projected onto the cm‐rep model for the tracts of interest in 3D, as shown in Figure 1: the corpus callosum (CC), cortico‐spinal tracts (CST), inferior fronto‐occipital fasciculi (IFO), inferior longitudinal fasciculi (ILF), superior longitudinal fasciculi (SLF), and uncinate fasciculi (UNC). To quantitatively compare and correlate the FA values with neurocognitive scores (Section 2.4), we calculated mean FA values for each subject in the atlas space in each tract.

### 2.4 Neurocognitive assessment

All participants completed a three‐ to four‐hour battery of standardized psychometric measures. From this battery, we chose to examine measures directly related to white matter integrity: processing speed (i.e., the speed of information processing), working memory (i.e., the ability to briefly hold and manipulate information in one's mind) and executive functions (i.e., higher level cognitive skills that involve mental control and self‐regulation). Executive functions assessed included inhibition (i.e., the ability to resist prepotent or impulsive responses), cognitive flexibility (i.e., the ability to shift one's focus within or between tasks), and phonemic and semantic verbal fluency (i.e., rapidly generating words beginning with particular letters or in specific categories). For participants on chronic blood transfusions, testing was performed within 1 week of transfusion to minimize fatigue effects. Additionally, the NIH PROMIS fatigue scale was given to all participants to screen for fatigue.

Testing was performed by the study neuropsychologist or by doctoral trainees under the supervision. Working memory was assessed with Digit Span from the Wechsler Intelligence Scale for Children, Fourth Edition (WISC‐IV) (Wechsler, 2003) or the Wechsler Adult Intelligence Scale, Fourth Edition (WAIS‐IV) (Wechsler, 2008), processing speed with Coding and Symbol Search from the WISC‐IV or WAIS‐IV, and executive functioning with the Color‐Word Interference, Trail Making and Verbal Fluency subtests of the Delis‐Kaplan Executive Function System (D‐KEFS) (Delis et al., 2001).

### 2.5 Statistical analysis

To compare the demographic characteristics and psychometric performance between the groups, two‐sample *t* tests and Pearson's *X* ^2^ tests were used for continuous and categorical variables, respectively. Bonferroni correction was used to correct for multiple comparisons. All neurocognitive tests were scored using the non‐medical, age‐adjusted normative data provided by the test publishers. To correct for the effect of outliers, we performed a nonparametric, one‐way Wilcoxon Rank test on the results using JMP (JMP^®^12.1.0).

Statistical differences in FA between the patient groups and healthy controls were assessed using a supra‐threshold statistical model (Yang et al., n.d.). A two‐sample *t* test at each point on the skeleton surface of a tract was computed. An arbitrarily chosen *t*_0_ was set to extract clusters on the surface for which t values are less than *t*_0_. The mass of each cluster (the area of the cluster in this context) was compared to a histogram of maximal cluster, which was computed from a large number of identical experiments where the labels of the subjects are randomly permuted. This comparison yields a *p*‐value for each cluster, and the histogram of cluster masses is pooled over all tracts. To correct for the family‐wise error rate (FWER), we used a permutation‐based cluster analysis method (Nichols & Holmes, 2002), taking into account the number of WM tracts. The threshold *p*‐value was set to .01 and the number of permutations to 10,000. We included age and sex in the general linear model in the TSA pipeline to control for relevant confounding factors.

To explore the quantitative FA value differences between groups, one‐way ANOVA with repeated measures was performed on the FA values of the 11 tracts between the tree groups: SCD, ACTL and controls. To investigate how WMH would affect FA within SCD group, we compared FA values of the 11 WM tracts of a subgroup of SCD patients with WMHs (without removing the voxels of FA maps that corresponding to the hyperintense‐voxels on T2‐FLAIR) and without WMHs to healthy controls, respectively. Finally, for the SCD group FA values were correlated for the 11 tracts with the nine study measures of neurocognitive performance, using the Benjamini–Hochberg procedure (Benjamini & Yoav, 2006), to decrease the false discovery rate.

## 3 RESULTS

### 3.1 Basic clinical characteristics of participants

Participants' demographic information, WMHs, and neurocognitive test performance are summarized in Table 1. SCD participants (*N* = 26) were African American or white Hispanic, ages 24.2 ± 9.7 years old with a balanced number of males and females (*F* = 13, *M* = 13). Four of the 26 SCD patients were on chronic transfusion therapy for this indication. The control group did not demonstrate significant differences in age and sex with participants with SCD. Twelve control participants were first‐ or second‐degree relatives of the SCD patients. There was a non‐significant gender imbalance (*p* > .05) compared to SCD group. To control for possible changes related to anemia, independently of sickle hemoglobin, we also recruited 19 participants with non‐sickle chronic anemia syndromes (ACTL), including beta‐thalassemia major (*N* = 11), beta‐thalassemia intermedia (*N* = 7), and congenital dyserythropoietic anemia (*N* = 1). ACTL age (26.1 ± 11.7) and sex (*F* = 10, *M* = 9) distributions were comparable to those of the SCD patients. Fourteen of the 19 ACTL patients were receiving chronic transfusion therapy. Due to relatively more various types of anemia, it was not possible to ethnically match the ACTL and SCD populations.

**TABLE 1:** Demographical characteristics and neuropsychological performance results

Demographics	CTL (*N* = 21)	SCD (*N* = 26)	*p*‐value	CTL (*N* = 21)	ACTL (*N* = 19)	*p*‐value
Age (mean ± *SD*)	22.6 ± 8.9	24.2 ± 9.7	.5	22.6 ± 8.9	26.1 ± 11.7	.4
Female	13 (62%)	13 (50%)	.7	13 (62%)	8 (42%)	.8
Hemoglobin^b^	13.4 ± 1.3	9.5 ± 2.0	<.0001	13.4 ± 1.3	10.5 ± 1.9	<.0001
Hematocrit^b^	40.1 ± 3.5	28.9 ± 4.9	<.0001	40.1 ± 3.5	32.3 ± 6.0	<.0001
Hemoglobin A (%)^b^	82.7 ± 17.8	22.8 ± 33	<.0001	82.7 ± 17.8	91.9 ± 7.4	.04
No. transfused (%)	0	8 (31%)	—	0	13 (68%)	—
No. participants with WMHs	7	14	.4	7	8	.8
Distribution of WMHs in most affected regions (L/R)	Frontal (47/39)		Parietal (8/10)		Temporal (2/2)	
Percentage (L/R)	29.5%/35.6%		6.0%/7.5%		1.5%/1.5%	

Neuropsychological tests^a^	CTL (*N* = 21)	SCD (*N* = 26)	*p*‐value	CTL (*N* = 21)	ACTL (*N* = 9)	*p*‐value^c^
FSIQ	104.5 ± 11.6	91.5 ± 12.1	0.03	104.5 ± 11.6	99.7 ± 9.8	—
Digit span	9.4 ± 3.3	9.4 ± 3.0	0.9	9.4 ± 3.3	9.6 ± 3.0	—
Coding^b^	9.1 ± 2.9	7.2 ± 2.8	0.03	9.1 ± 2.9	9.7 ± 3.4	—
Symbol search^b^	10.0 ± 2.1	8.6 ± 2.2	0.03	10.0 ± 2.1	9.8 ± 2.3	—
Inhibition	10.4 ± 3.1	8.4 ± 3.2	0.05	10.4 ± 3.1	10.0 ± 3.0	—
Inhibition/Switching	10.1 ± 1.7	8.9 ± 3.1	0.08	10.1 ± 1.7	10.1 ± 1.6	—
Letter fluency	11.0 ± 3.8	8.9 ± 3.6	0.06	11.0 ± 3.8	9.8 ± 2.7	—
Category fluency	9.6 ± 1.7	8.2 ± 3.9	0.2	9.6 ± 1.7	9.9 ± 1.9	—
Switching	9.9 ± 3.5	8.7 ± 2.9	0.3	9.9 ± 3.5	10.3 ± 3.1	—
Trail making test	9.4 ± 3.5	9.1 ± 3.0	0.8	9.4 ± 3.5	9.5 ± 2.3	—

Abbreviations: ACTL, anemic controls; CTL, controls; FSIQ, full‐scaled IQ; L/R, left hemisphere/right hemisphere; SCD, sickle cell disease; WMH, white matter hyperintensity.

^a^ Psychometric scores are presented as scaled scores, which have a mean of 10 and a standard deviation of 3, followed by the range.

^b^ Significant difference between two groups using *t* test with Bonferroni correction.

^c^ Neuropsychological tests are only available for nine patients in ACTL group; therefore, they are not analyzed.

### 3.2 Tract‐specific analysis

**FIGURE 2:**
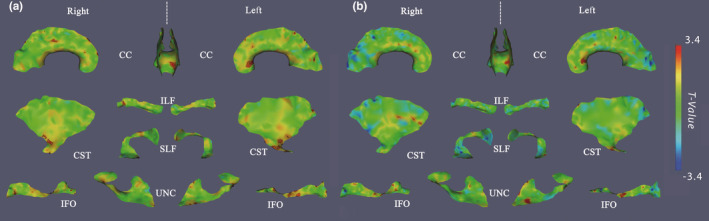
The significant clusters of reduced FA in patients with SCD (panel a) and non‐sickle anemic syndromes (panel b) compared to healthy controls (in red), overlaid on the corresponding *t*‐statistics maps on the skeleton surfaces of 11 tracts

We observed lower FA in most of the 11 tracts tested in SCD patients compared with healthy controls (Figure 2a). In each group comparison, red indicates lower FA in SCD in comparison to controls and blue indicates lower FA values in controls; none of the blue regions approached significance.

The most pronounced differences in FA between SCD patients and healthy controls are found along the CC. The largest cluster along the CC is found on the genu on the left hemisphere. Noticeable areas of difference observed bilaterally along the CST are mostly located inferiorly. The IFO shows larger and more numerous regions of significant difference on the left hemisphere in comparison to the right. Significant FA differences were observed on the lateral portion of the right ILF, SLF, and left UNC while no significant FA differences were discernible on either the left ILF or the left SLF between SCD patients and controls. All the significant FA different areas are shown in red clusters in Figure 2a.

To determine the impact of anemia alone on WM integrity, Figure 2b shows FA comparison of 11 WM tracts between the ACTL and CTL groups. The clusters on the right CST and right ILF in Figure 2a disappear in Figure 2b. The significant areas on left genu of the CC are also seen in ACTL with CTL, as well as that on the left IFO.

**FIGURE 3:**
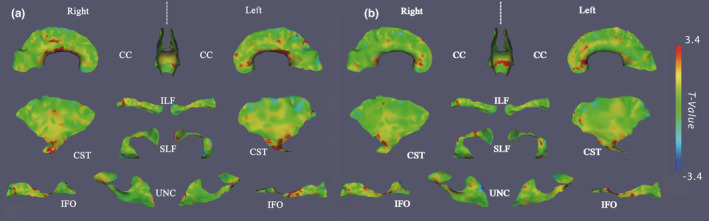
The significant clusters of reduced FA in SCD patients with white matter hyperintensities (panel a) and patients without hyperintensities (panel b) compared to healthy controls (in red), overlaid on the corresponding *t*‐statistics maps on the skeleton surfaces of 11 tracts

From the T2‐FLAIR images, white matter hyperintensities (WMHs) were observed in 14 out of 26 subjects in the SCD group by two on‐site raters in consensus (one Ph.D. student with a biomedical imaging background and one medical graduate student). The number and location of each WMH were confirmed by an experienced board certificated neuroradiologist, as described in detail in a previous study (Xu et al., 2018), with accumulated WMHs of 132 distributed mostly in frontal and parietal lobes (Table 1). While the lesions were typically quite small and were not expected to affect the tractography, we performed quality control on the tracts generated by the TSA to ensure their integrity, particularly around the lesions. Figure 3 demonstrates FA differences in the subgroup of SCD patients with WMHs (Figure 3a) and without WMHs (Figure 3b) compared to CTL. The patterns are similar in both comparisons for ILF, SLF, UNC, and IFO. However, when we directly compared the patients with WMHs against the ones without WMHs, there were no significant clusters on the inferior portion of CST or the left genu of CC. The only significant clusters were observed on the anterior potion of the left SLF, of which the location and size are consistent with the ones observed in Figure 3a; therefore, the figure was not included.

In addition, we compared mean FA values of each tract in each subject between the SCD, ACTL, and CTL groups, after masking the FA maps with the tracts in the atlas space. Mean FA values of the CC and right CST are significantly lower in SCD than the ones in controls. In comparison, only mean FA in the CC (*p* = .007) and the right CST (0.042) showed a significant difference between in the repeated one‐way ANOVA test.

### 3.3 Neurocognitive data analysis and correlation with neuroimaging

Given the prevalence of FA abnormalities observed in Figure 2, we compared neurocognitive performance between the SCD and CTL subjects. Neurocognitive testing was only completed in six of the 19 ACTL subjects, who additionally were not comparable in terms of race or parents' education and income level to either the CTL or SCD groups. Therefore, the ACTL group was not included in neurocognitive analysis. While the SCD group had lower scores in all but one metric, the only statistically significant group differences were found on Symbol Search (*p* = .027) and Coding (*p* = .030), both measures of processing speed and Inhibition (*p* = .053), a measure of response inhibition. A non‐significant trend was suggested for Letter Fluency, a lexical verbal fluency task (*p* = .061). No significant group differences were found for Category Fluency (semantic verbal fluency), Digit Span (working memory), Color‐Word Interference (response inhibition), and Trail Making (cognitive flexibility).

To examine the potential relationship between neurocognitive performance and WM microstructural alterations in the SCD group, we performed correlations for each neurocognitive test by mean FA values in each of the 11 tracts. Only the FA changes in CC were associated with the neurocognitive data, and significant correlations were observed across all nine neurocognitive measures (despite correction for multiple comparisons): Processing Speed: Symbol Search (*p* = .012, *r* = .30) and Coding (*p* = .006, *r* = .34); Working Memory: Digit Span (*p* = .011, *r* = .27); Response Inhibition: Inhibition (*p* = .007, *r* = .51); Cognitive Flexibility: Inhibition/Switch (*p* = .007, *r* = .57), Trail Making (*p* = .011, *r* = .29), and Verbal Fluency: Category Switch (*p* = .011, *r* = .25), and lexical (Letter, *p* = .037, *r* = .23) and semantic (Category, *p* = .015, *r* = .24) verbal fluency.

## 4 DISCUSSION

We examined the differences in 11 major WM tracts between patients with SCD, non‐sickle anemic controls, and healthy controls, using the most widely used DTI metric: FA. FA has been widely used in tensor‐derived measures and is modulated by axonal density, axonal caliber and the degree of myelination. (Klingberg & Vaidya, 1999; Schmithorst et al., 2002; Ulu, 2002). The results were calculated using a medial representation of tracts, and a deformable shape analysis technique, TSA, which directly projects areas of significant differences between WM tracts onto surfaces (Zhang et al., 2010). Additionally, we analyzed the neurocognitive performance of the SCD patients and healthy controls and further examined the relationship between FA values and neurocognitive performance in SCD. To investigate the impact of anemia alone on WM microscopic changes, we also compared FA differences between ACTL and healthy controls.

SCD patients demonstrated widespread lower WM FA on each of 11 tracts except the SLF and UNC. Many of the changes observed in the SCD group were also observed in the ACTL group, but in smaller size and with a sparser distribution, especially in the CC, and the right and left CST. Reduced FA simply means that the diffusion orientation‐dependent aspects of the microstructure of the WM are different in anemic subjects, but further studies are needed to associate the FA findings with specific anomalies in the underlying tissue microstructure. In view of the pathology literature, it suggests that sickle cell anemia is associated with acute demyelination (Kimmelsteil, 1948). Another study using TBSS in SCD patients indicates that both axonal loss and demyelination contribute to the reduced FA (Kawadler et al., 2015). However, without additional experiments specifically probing these questions, it is too soon to draw further interpretations.

The corpus callosum (CC), the largest and most prominent WM structure in the brain, connects the left and right cerebral hemispheres and consists of more than 200 million axonal projections to the various parts of the cerebral cortex. Our observed decrease in FA parallels the structural alterations of the CC, including shape deformation and reduced FA specific to genu (Balci et al., 2012; Sun et al., 2012). It has been demonstrated that WM has impaired oxygen transport in patients with either sickle or non‐sickle anemic syndromes (Chai et al., 2019). This chronic hypoxic condition may irreversibly reduce the extent of myelination in the CC, as suggested in a study in mice (Kanaan et al., 2019). However, the effect of chronic hypoxia on myelination of the CC, or more generally, longitudinal WM alterations due to anemia or hypoxia, has not yet been studied in humans. Nevertheless, the reduced FA in the genu, possibly reflecting synaptic pruning and dysmyelination at the microscopic neuronal level, could be one of the consequences of chronic hypoxia on WM.

Changes in the inferior right CST appear unique to SCD patients in our overall sample and may be related to the pathophysiology mechanism of sickled hemoglobin. The CST interconnects the motor cortex with the brainstem and spinal cord, thus playing a vital role in controlling muscular movements in the body. Our results for the CST are consistent with the results in Balci et al. (2012), who observed fewer fiber counts on both left and right CST using ROI‐based analysis. The clusters on the inferior portion imply degenerated cortico‐spinal pathways, which would suggest an impact on motor functioning. While motor functioning was not assessed in our study, obvious motor deficits were not observed in our participants, consistent with previous reports in patients with SCD with no overt neurological symptoms (Balci et al., 2012; Vichinsky et al., 2010).

On the other hand, changes in the IFO were found in both SCD and ACTL patients. The IFO is a prominent WM tract that connects the frontal lobe with the occipital cortex and temporo‐basal areas (Martino et al., 2010). Due to its connectivity, the functional influences are as follows: semantic elaboration of language for the superficial layer and the posterior region of the deep layer; integration of multimodal sensory inputs and motor planning functions for the middle region; emotional and behavioral impacts for the anterior region (Sarubbo et al., 2013). The left middle portion of IFO in Figure 2a,b both show significant difference, which indicates that these areas are affected by anemia in general, regardless of phenotype.

Consistent with van der Land et al. (2016), we found the presence or absence of WMHs was a marker of disease severity in the SCD group, but this did not fundamentally alter the distribution of significant areas of FA abnormality: reduced FA present on Figure 2a was also present in patients with WMHs (Figure 3b). The significant areas of CC and SLF in Figure 3b are more widely distributed than Figure 3a; however, we did not observe a significant difference when directly comparing patients with WMH against patients without WMH within SCD group. On the contrary, the patterns in the CST, left ILF, and IFO are reversed: significant differences are less discernible in Figure 3b than those of Figure 2a. Given that the WMHs were distributed mostly in frontal (65%) and parietal lobe (14%), the FA changes were found in varying locations across different patients, which suggests that they are biomarkers of more diffuse WM disease, independent on the location of the WMH. That is, WMH represents an iceberg phenomenon, having microscopic structural damage far exceeding the areas exhibiting WMH on T2‐FLAIR images. The significant lower FA regions on the genu of the CC and the anterior region of IFO, ILF, and left SLF in both Figure 2a and three may indicate the vulnerability of the WM in the frontal and parietal lobes, which are the regions with the lowest cerebral blood flow (Chai et al., 2019; Ford et al., 2018). Our findings are consistent with Ford et al. (2018), who reported a large SCI density in the pediatric patients with SCIs, in the frontal lobes (90%), followed by the parietal lobes (53%) in a multi‐center pediatric SCD study.

Regarding neurocognitive test findings in SCD patients, we found performance was significantly lower on measures of processing speed in comparison to healthy controls, in line with previous reports in adults with SCD who have no known history of overt stroke (Balfour, 2018; Crawford & Jonassaint, 2015; Schatz et al., 2018; Steen et al., 2003; Vichinsky et al., 2010). Processing speed is a basic neurocognitive process sub‐serving other cognitive functions (Viana‐Baptista et al., 2008). In its broadest sense, it is defined as the speed at which one can perform mental operations, though many measures also involve a fine motor component. Processing speed is a critical component in the acquisition of new learning, as well as in the efficient retrieval and integration of previous learning (Brébion et al., 2007). In addition to its impact on cognitive domains such as learning and retrieval, processing speed tests themselves are often a component of Full Scale Intelligence Quotients (FSIQ). In addition to verbal and nonverbal reasoning, FSIQ may include working memory and processing speed, depending on the measure. This would partially account for the lower FSIQ scores reported in studies of SCD that used intelligence measures that included processing speed (Brown et al., 2007; Daniel Armstrong et al., 1996; Kral & Brown, 2004; Schatz et al., 2001, 2002).

Deficits in processing speed can stem from focal or diffuse injuries to the white matter, as well as from subcortical lesions, for example, in the caudate (Righart et al., 2013). Processing speed deficits are commonly seen with axonal injury in traumatic brain injury (Felmingham et al., 2004), following cranial irradiation for cancer treatment (Askins and Moore, 2008; Schatz, et al., 2001) and in demyelinating disorders such as multiple sclerosis (Barker‐Collo, 2006; Demaree et al., 1999; Genova et al., 2009). Our finding of lower processing speed in SCD patients with no known history of overt stroke is consistent with microscopic structural damage to WM tracts. Additionally, our SCD group's neurocognitive performance was also significantly lower, in comparison to healthy controls, on a measure of inhibition. Inhibition is an executive function involving resisting a prepotent, overlearned, or impulsive response. Inhibition and other psychometric measures of executive functions are timed, and therefore, performance is mediated by processing speed. Deficits in inhibition have been associated with WM integrity (Bessette & Stevens, 2019; Hinton et al., 2018) and SCD (Daly et al., 2014; Hijmans et al., 2011). More broadly, deficits in executive functions, including working memory, have been reported in SCD (Crawford & Jonassaint, 2015; Daniel Armstrong et al., 1996; Mackin et al., 2014).

In our examination, we found that lower FA values along the CC were significantly associated with lower neurocognitive performance in our SCD group. Significant correlations were not indicated for the other tracts, likely due to less spatial sensitivity to capture potential relationships (Tournier et al., 2011). Given the CC is by far the largest commissural tract responsible for communication between the left and right hemispheres, the integrity of its axons would play a critical role in the speed of information transfer. Regarding our significant findings between FA values and measures of executive functions, the genu of the CC has fibers which connect the prefrontal cortices of the two hemispheres while the orbital‐frontal cortices are connected by fibers from the rostrum of the CC (Goldstein & Mesfin, 2017). The relationship between the prefrontal cortex and executive functions has been examined since 1848, when Dr. Harlow began treating and studying Phineas Gage after an industrial accident sent an iron rod through Gage's left frontal lobe, leading to significant change in his personality and behavior (O'Driscoll & Leach, 1998). Of note is the orbital‐frontal aspect of Gage's injury and his subsequent inability to inhibit behavior (O'Driscoll & Leach, 1998). More recent studies (Alvarez & Emory, 2006; Damasio & Anderson, 1993; Yuan & Raz, 2014) have elucidated the role of the prefrontal cortex as the primary coordinating region for executive functions, with connections to diverse brain regions involved in specific executive functions. The role of the CC in transferring information between the hemispheres, as well as between the prefrontal cortices, is consistent with our significant findings supporting the role of CC integrity and functional outcomes in processing speed and executive functions in SCD.

Our study has several limitations. Neuronal connections of WM extend beyond the continuous medial representation of the sheet‐like 11 tracts, if observed using tractography (Kamagata et al., 2013). TSA is not able to reveal the full WM connections, especially in the distal areas of deep WM structures, which explains the very small clusters shown on the edge of right SLF, right CC in Figure 2a and left UNC in Figure 2b. In addition, the diffusion tensor model itself is less capable of evaluating FA values in regions with crossing fibers, thereby potentially underestimating FA values in the regions where fiber kissing, curving, and branching are pronounced. Hence, the next stage of our work will be to employ alternative acquisition protocols and modeling methods to remedy this problem. It will be beneficial in the future to rescan our study participants with multiple High Angular Resolution Diffusion Imaging (HARDI) shells, to allow a better estimation of single fiber orientation and resolution of multiple crossing fibers. Secondly, several new processing methods have also been designed to estimate fiber orientations in crossing regions (Caan et al., 2010). Those typically work better for HARDI acquisitions than for ours and use for instance spherical deconvolution‐based methods such as hindrance modulated orientational anisotropy (Dell'Acqua et al., 2013), or more recently, Cabeen et al. (2016) proposed a model‐based kernel regression framework for estimating fiber orientation mixtures. Finally, TSA uses deterministic tractography. In future work, we will also make use of probabilistic tractography methods (Mishra et al., 2015).

In terms of our study group, our SCD patients are heterogeneous with respect to genotype and treatment (e.g, blood transfusion status, hydroxyurea), as are our ACTL patients. While we were able to obtain a broad range of hemoglobin values (by study design), we did not have the statistical power to characterize the impact of transfusions, hydroxyurea, or genotype, nor the power to compare the neurocognitive performance differences between SCD patients with WMH and without(Balfour, 2018). Despite this, microstructural differences between our anemic and non‐anemic participants were observed and in line with previous work, highlighting the sensitivity of the TSA methods.

In conclusion, our study is the first to investigate the impact of anemia and sickled hemoglobin separately on the microstructural changes in the 11 major WM tracts. Decreased FA in the genu of CC, left inferior CST and IFO was observed in chronically anemic patients, regardless of anemia subtype, while FA reductions in the CST and SLF were unique to SCD patients. Patients with WMHs had more significant FA abnormalities, which were found in the same areas as patients without WMHs. Slower processing speed and response inhibition skills were observed in SCD patients, consistent with WM involvement. Decreased FA values in the CC significantly correlated with all nine neurocognitive measures, which included processing speed, working memory, and executive functions, suggesting a critical importance for CC fiber integrity in core neurocognitive processes. Future work on this study will include examining the thickness and the integrity of WM interconnections, to further understand WM damage and neurocognitive functioning in SCD patients.

## CONFLICT OF INTEREST

JCW receives research support in kind from Philips Healthcare. None of the other authors have conflicts relevant to the study.

## AUTHOR CONTRIBUTION

JCW and TC are the principal investigators of the overall project. They recruited subjects and JCW collected imaging data. BT supervised lesion identification, which was performed by YC. YC, CJ, JCW, SHO, and NL designed and conducted the study, analyzed the data, and wrote the manuscript. MCB assisted with data analysis and manuscript writing. SC and CV helped with data analysis.

### Peer Review

The peer review history for this article is available at https://publons.com/publon/10.1002/brb3.1978.

## Data Availability

De‐identified imaging and clinical data from this manuscript will be made available to qualified scientific person for investigative purposes contingent upon approval from legal and regulatory authorities at Children's Hospital of Los Angeles.
